# NMR studies on the interactions between yeast Vta1 and Did2 during the multivesicular bodies sorting pathway

**DOI:** 10.1038/srep38710

**Published:** 2016-12-07

**Authors:** Jie Shen, Zhongzheng Yang, Jiaolong Wang, Bin Zhao, Wenxian Lan, Chunxi Wang, Xu Zhang, Cody J. Wild, Maili Liu, Zhaohui Xu, Chunyang Cao

**Affiliations:** 1 State Key Laboratory of Bioorganic and Natural Products Chemistry and Collaborative Innovation Center of Chemistry for Life Sciences, Shanghai Institute of Organic Chemistry, Chinese Academy of Sciences, 345 Lingling Road, Shanghai, 200032, China; 2 Tianjin Institute of Industrial Biotechnology, Chinese Academy of Sciences, 32 XiQiDao, Tianjin Airport Economic Area, Tianjin, 300308, China; 3 Public Technology Service Platform, Wuhan Institute of Biotechnology, 666 Gaoxin Road, Wuhan, 430075, China; 4 State Key Laboratory of Magnetic Resonance and Atomic and Molecular Physics, Wuhan Institute of Physics and Mathematics, Chinese Academy of Sciences, 30 West Xiaohongshan Road, Wuhan, 430071, China; 5 Life Sciences Institute and Department of Biological Chemistry, Medical School, University of Michigan, 210 Washtenaw Ave, Ann Arbor, MI 48109, USA

## Abstract

As an AAA-ATPase, Vps4 is important for function of multivesicular bodies (MVB) sorting pathway, which involves in cellular phenomena ranging from receptor down-regulation to viral budding to cytokinesis. The activity of Vps4 is stimulated by the interactions between Vta1 N-terminus (named as Vta1NTD) and Did2 fragment (176–204 aa) (termed as Did2_176–204_) or Vps60 (128–186 aa) (termed as Vps60_128–186_). The structural basis of how Vta1NTD binds to Did2_176–204_ is still unclear. To address this, in this report, the structure of Did2_176–204_ in complex with Vta1NTD was determined by NMR techniques, demonstrating that Did2_176–204_ interacts with Vta1NTD through its helix α6′ extending over the 2^nd^ and the 3^rd^ α-helices of Vta1NTD microtubule interacting and transport 1 (MIT1) domain. The residues within Did2_176–204_ helix α6′ in the interface make up of an amino acid sequence as E_192_′xxL_195_′xxR_198_′L_199_′xxL_202_′R_203_′, identical to type 1 MIT-interacting motif (MIM1) (D/E)xxLxxRLxxL(K/R) of CHMP1A_180–196_ observed in Vps4-CHMP1A complex structure, indicating that Did2 binds to Vta1NTD through canonical MIM1 interactions. Moreover, the Did2 binding does not result in Vta1NTD significant conformational changes, revealing that Did2, similar to Vps60, enhances Vta1 stimulation of Vps4 ATPase activity in an indirect manner.

The ESCRT (endosomal sorting complexes required for transport) machinery^1^,^2^,^3^, initially identified in the yeast multivesicular bodies (MVBs) biogenesis pathway^3^,^4^, protects against age-related neurodegenerative diseases through either the canonical MVB pathway^5^,^6^,^7^ or autophagy^8^,^9^, and plays a pathological role in viral infection^6^,^10^,^11^. It is made up of ESCRT-0, ESCRT-I, ESCRT-II, ESCRT-III and Vps4 complexes^12^,^13^. ESCRT-0 is responsible for clustering of ubiquitylated cargoes to the site of MVB formation. ESCRT-I and ESCRT-II together generate membrane curvature and bud while assembly of ESCRT-III at the bud neck catalyzes the scission of membrane. These components have been identified as potential tumor suppressors^8^, mainly due to the involvement of the ESCRT machinery in regulating signal attenuation for activated receptors of growth factors, peptide hormones and cytokines. Vps4 is an AAA-ATPase, which disassembles ESCRT-III polymers upon ATP binding and hydrolysis^14^,^15^.

The biogenesis of lysosomes involves the maturation of early endosomes into MVBs. During this pathway, portions of limiting membrane of the endosome invaginate and then detach into lumen of endosome, forming intraluminal vesicles (ILVs)^6^,^7^. Then MVBs fuse with the lysosome and ILVs, thus their components are degraded. The cell surface receptors for down-regulation and some enzymes located in lysosomes are sorted into this endo-lysosomal degradation pathway^16^. The ATP consuming reaction is the only step in MVB biogenesis that inputs energy to the system, therefore providing the thermodynamic driving force for disassembly process. The function of Vps4 is conserved in all biological processes that depend on the action of the ESCRTs. Identical to other AAA-ATPases, Vps4 acts as an oligomer with two conformationally distinctive hexameric rings^17^. These two rings have a central pore, so that ESCRT-III subunits may physically interact Vps4 and pass through this pore during the disassembly process. Vps4 interacts with ESCRT-III subunits through its N-terminal microtubule-interacting and transport (MIT) domain^18^, which appears to specifically identify short peptide sequence MIT-interacting motifs (MIMs) at or near the C-terminal end of ESCRT-III subunits^19^,^20^,^21^,^22^,^23^,^24^.

Vta1, Did2, Ist1 and Vps60 are ESCRT-III related proteins, which have been identified to bind to Vps4 and play key roles in regulating the oligomerization and activity of Vps4 *in vivo*^25^,^26^,^27^,^28^,^29^. Vta1 is a positive regulator of Vps4 by promoting Vps4 oligomerization^26^,^30^. Based on its structural studies, Vta1 is a molecular dimer with each subunit folded into two terminal domains linked by a flexible linker^29^. Its C-terminal domain mediates dimerization and binds to a unique β-domain in the Vps4 AAA domain^31^,^32^. Its N-terminal domain (residues 1–167 and was named as Vta1NTD in this report, Fig. 1A) contains two tandem MIT domains, which specifically recognize Vps60 and Did2^27^,^29^. The fragment Vps60 (128–186aa) (termed as Vps60_128–186_) was reported to display full activity of Vps60, which stimulates Vps4 ATPase in a Vta1-dependent manner^27^. Our NMR structural studies on the interactions between Vta1NTD and Vps60_128–186_ revealed that Vps60_128–186_ interacted with Vta1NTD in a novel MIT recognition mode, *i.e.*, through its helices α4′ and α5′ extending over helices 5, 6 and 7 of Vta1NTD MIT2 domain^33^,^34^,^35^,^36^. Previous studies indicated that the C-terminal of Did2, residues 176–204, named as Did2_176–204_ in this whole report, as shown in Fig. 1A, could enhance Vps4 ATPase activities as the full-length Did2^27^,^29^. In this report, to investigate how Did2 interacted with Vta1NTD, we first measured the binding affinity of Vta1NTD to Did2_176–204_ by isothermal titration calorimetry (ITC) assay (*K*_D_ = 12.8 ± 1.0 μM, the number N = 1.16 ± 0.0266) (Fig. 1B), and then determined the solution structure of Vta1NTD in complex with Did2_176–204_. This structure revealed that Did2_176–204_ bound to Vta1NTD through canonical type 1 MIT-interacting motif (MIM1) interactions.

## Results and Discussion

### The suggested MIM region of Did2_176–204_ forms an α-helix conformation

**Figure 1:**
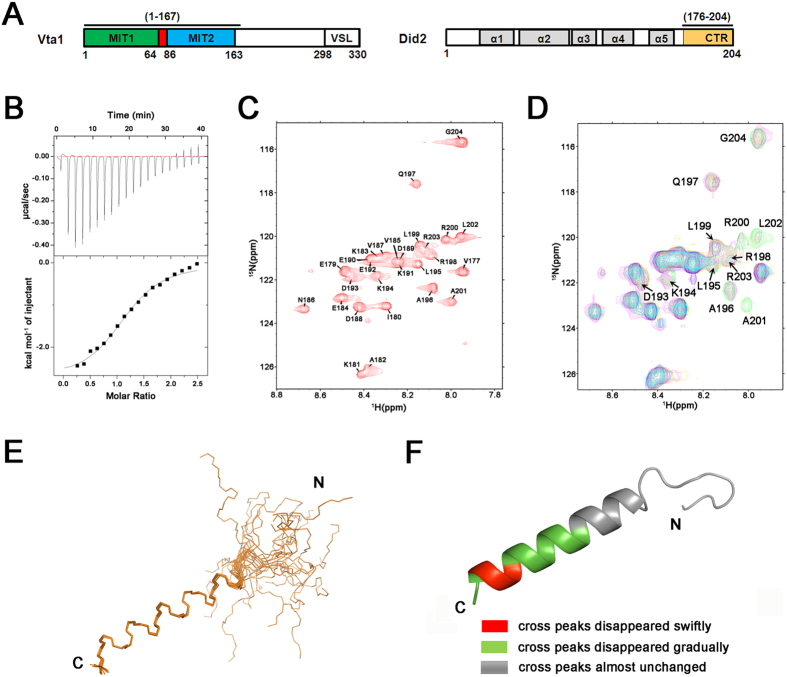
Did2_176–204_ MIM interacts withVta1NTD. (**A**) Schematic representation of Vta1 and Did2, highlighting regions critical for interactions that contribute to increased Vps4 ATPase activity. (**B**) The binding affinity of Did2_176–204_ to Vta1NTD, measured by ITC assay. Upper panel shows the raw data for the titration of Did2_176–204_ with Vta1NTD and lower panel displays the heats of binding integrated from the peaks. The solid line in the bottom panel represent the best curve fit to the experimental data. (**C**) NMR signal assignments of the cross-peaks in the ^1^H-^15^N HSQC spectrum of free Did2_176–204_. (**D**) NMR titration of Vta1NTD into Did2_176–204_ at mole ratios (Did2_176–204_ *vs* Vta1NTD) of 1:0 (green), 1:0.1 (red), 1:0.2 (golden), 1:0.4 (blue), 1:1.2 (cyan). The residues D193-G204, corresponding to the residues marked in red and green in Fig. 1F, whose cross peaks finally disappeared during titration, were highlighted. (**E**) The backbone view of the ensemble of 20 lowest-energy free Did2_176–204_ structures. (**F**) Ribbon representation of free Did2_176–204_, the residues whose cross-peaks disappeared swiftly, disappeared gradually and almost unchanged were highlighted in red, green and grey colors, respectively.

Secondary structure prediction and perceived structural homology to ESCRT-III protein Vps24/CHMP3 suggest that Did2_176–204_ corresponds to the 6^th^ helix within the Did2 structure^37^. This helix conformation was confirmed by our following NMR studies. We first assigned its NMR signals based on two-dimensional (2D) ^1^H-^1^H TOCSY and NOESY, three-dimensional (3D) ^15^N-edited HSQC-TOCSY spectra. In its 2D NMR ^1^H-^15^N HSQC experiment (Fig. 1C), the cross peaks belonging to 27 residues were assigned, except the N-terminal residues N_176_ and P_178_ (without amide proton). We then determined its NMR solution structure using 191 distance restraints from NOE and 26 hydrogen bonds. Finally, 20 structures with the lowest-energy could be well fitted (Fig. 1E) with the RMSD values of 0.15 ± 0.06 Å for the backbone atoms, and of 0.88 ± 0.09 Å for all heavy atoms in secondary structure region. The Ramachandran plot displays 93.1% of the residues in the most-favored regions, 4.0% residues in additionally allowed regions, and 2.9% residues in generously allowed regions, indicating that the structures are acceptable. The solution structures demonstrate that the region of Did2 187–204 aa forms an α-helix, consistent with the observation in the crystal structure of complex Did2-Ist1^27^,^29^.

To study how Vta1NTD interacts with Did2_176–204_, we performed NMR titration experiments, in which Vta1NTD was titrated into Did2_176–204_ at mole ratios (Vta1NTD *vs* Did2_176–204_) of 0.1:1, 0.2:1, 0.4:1, 0.6:1, 0.8:1 and 1.2:1 in NMR buffer conditions. The cross peaks belonging to residues R_200_A_201_L_202_ disappeared even at mole ratios of 0.1:1 and 0.2:1, while the cross peaks belonging to D193-L199 and R_203_G_204_ became weaker and weaker upon the concentration of Vta1NTD being increased. At the mole ratio of 1.2:1, the cross peaks belonging to residues V177-E192 almost unchanged (Fig. 1D). These observations suggest that the residues D193-G204 are involved in the interactions, and that the MIM sequence of Did2_176–204_ bind to Vta1NTD. This hypothesis was consistent with our observation in the complex structure (discussed below), where the residues in the region of 187–204aa interact with Vta1NTD MIT1 domain.

### NMR structural determination of complex Vta1NTD-Did2_176–204_

**Figure 2:**
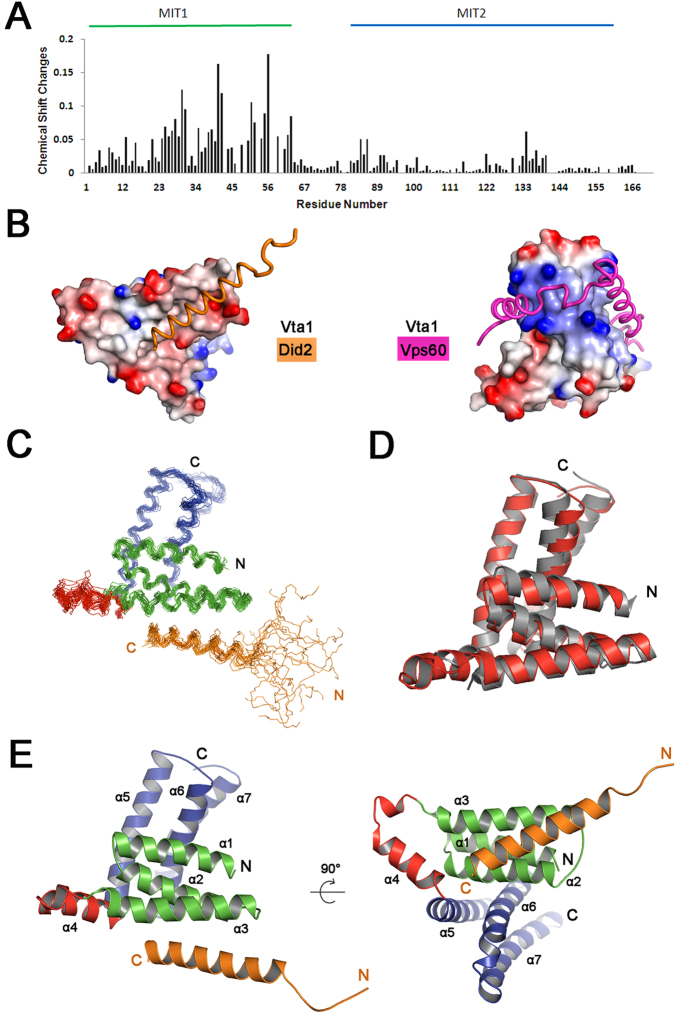
Did2_176–204_ interacts with helices 2/3 of Vta1NTD MIT1 motif. (**A**) The Chemical shift changes of Vta1NTD backbone atoms amide ^1^H and ^15^N upon Did2_176–204_ binding, calculated by using the equation: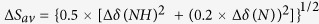. The MIT1 and MIT2 domains are indicated at the top based on the crystal structure of free Vta1NTD (PDB ID: 2RKK). (**B**) Differences in electrostatic potential surfaces between MIT1 and MIT2 of Vta1NTD. The electrostatic potential surface was generated based on the crystal structure of free Vta1NTD by using software DelPhi and visualized by PyMol. The Did2 and Vps60 were displayed in orange and pink tube mode, respectively. (**C**) Backbone view of the ensemble of 20 lowest-energy Vta1NTD-Did2_176–204_ NMR structures, where Did2_176–204_ is displayed in orange. (**D**) Structural overlay of free Vta1NTD (grey) (pdb code 2RKK) and bound Vta1NTD to Did2_176–204_ (red). The N and C termini and the secondary structures are indicated. (**E**) 3D representative structure of Vta1NTD-Did2_176–204_. The helices are numbered. In (**C**) and (**E**), the MIT1 and MIT2 domains of Vta1NTD, the linker between MIT1 and MIT2, and Did2_176–204_ were displayed in blue, green, red and orange colors, respectively.

Using two basic sets of NMR samples: 1) ^13^C and ^15^N isotope double labeled or ^13^C, ^15^N and 70% ^2^H triple-labeled Vta1NTD mixed with unlabeled Did2_176–204_ at the stoichiometric ratio of 1:1.2, 2) ^13^C and ^15^N isotope labeled Did2_176–204_ mixed with unlabeled Vta1NTD at stoichiometric ratio of 1:1.2, and by running a series of 2D and 3D NMR experiments, in total, more than 94% NMR signals of the main-chain and 95% NMR signals of the side-chain atoms of the residues in the complex were assigned. The inter-molecule NOEs were correctly assigned by confirming signals observed in 3D ^13^C-F1 edited, ^13^C/^15^N-F3 filtered NOESY spectra acquired on both complex samples. The NMR chemical shift changes of Vta1NTD backbone atoms ^1^H and ^15^N in the absence of and in the presence of Did2_176–204_ reveal that Did2_176–204_ addition mainly induces amide ^15^N and ^1^H chemical shifts variation of the residues in Vta1NTD MIT1 (Fig. 2A), suggesting that Did2_176–204_ binding sites locate in this region. This observation accords with the previous analysis on chemical shift mapping of CHIMP1B-binding site on LIP5(MIT)_2_^35^, and with the analysis of electrostatic surface of Vta1NTD, which shows that Vta1NTD MIT1 is more negatively charged and more hydrophobic than its MIT2 (Fig. 2B), suitable for positively charged and hydrophobic Did2_176–204_ binding.

The solution structure of Vta1NTD-Did2_176–204_ complex was then determined by a conventional heteronuclear NMR method using ^15^N-labeled or ^13^C/^15^N-labeled proteins. In total, 2730 distance restraints from NOE (including 36 inter-molecular NOEs), 236 hydrogen bonds and 676 dihedral angle restraints for backbone φ and ψ angles were used to calculate solution structure. The best-fit superposition of the ensemble of the 20 lowest-energy structures represented in Fig. 2C was displayed with the RMSD values of 0.82 ± 0.17 Å for the backbone (N, C_α_ and CO) atoms and 1.14 ± 0.15 Å for all heavy atoms in the well-ordered second structure regions. The Ramachandran plot displays 97.8% of the residues in the most-favored regions and 2.1% residues in additionally allowed regions (Table 1), indicating the structures are reasonable.

### Overall complex structure

The complex Vta1NTD- Did2_176–204_ structure shows that the bound Vta1NTD still has two MIT domains, each of them is composed of three α-helices (MIT1: helices α1, α2 and α3; MIT2: helices α5, α6 and α7; respectively), almost similar to those observed in its free state and in its complex with Vps60_128–186_^29^,^33^,^34^,^35^,^36^. The backbone atoms belonging to MIT1 and MIT2 regions of bound Vta1NTD have RMSD values of 1.7 Å and 1.8 Å with those of free Vta1NTD (Fig. 2D), respectively, indicating that Did2_176–204_ binding does not induce overall major conformational changes in Vta1NTD. The linker (64–85aa) between MIT1 and MIT2 domains is well ordered, where residues 66–69 become an α-helix, and residues 73–84 adopt a longer helical structure (here we called it as helix α4) (Fig. 2E)^33^,^34^. The helix α4 is much longer in Vta1NTD-Did2_176–204_ than that in free Vta1NTD crystal structure, but similar to the observation in NMR structure of Vta1NTD bound to Vps60_128–186_^33^,^34^,^35^. This observation is consistent with our previous secondary structure prediction of free Vta1NTD using the programs CSI^38^ and TALOS^39^,^40^ based on the assignments of NMR signals belonging to the backbone atoms of Vta1NTD. In contrast, this linker in free Vta1NTD crystal structure adopts largely random-coil structure with only a one-turn α-helix occurring at residues 80–84. Particularly, residues 65–75 appear to be disordered in the structure of free Vta1NTD. Thus, this conformational change might be caused by the interactions between helix α4 (73–84) of Vta1NTD and Vps60_128–186_ or Did2_176–204_, or by stacking during free Vta1NTD crystallization.

Within our expectation, in the current complex structure, the Did2_176–204_ folds into one α-helix (denoted as α6′ helix hereafter), which is involved in the interaction with the first MIT domain of Vta1NTD (Fig. 2E). The bound Did2_176–204_ adopts an overall rod-like helix structure with a flexible loop in its N-terminus (similar to free Did2_176–204_ with an RMSD value of 0.5 Å for the backbone atoms in secondary structure region). The helix consists of residues 187′–203′, a little longer than that (residues 184′–198′) observed in LIP5NTD-CHIMP1B complex structure^36^. The Did2_176–204_ α-helix sits on the surface groove formed by the 2^nd^ and the 3^rd^ helices of Vta1NTD MIT1 in a mode similar to that observed in Vps4-CHMP1A complex structure^20^ and that observed in human LIP5NTD-CHMP1B and LIP5NTD-CHMP1B-CHMP5 complex structures^36^. The Vta1-Did2_176–204_ complex buries a total of approximately 1039 Å^2^ surface area at the interface, close to that (1115 Å^2^) observed in the complex structure of Vps4-CHMP1A^20^, but much larger than that (~624 Å^2^) observed in the complex structures of LIP5NTD-CHMP1B and LIP5NTD-CHMP1B-CHMP5^36^.

### The interface in Vta1-Did2_176–204_ complex structure

**Figure 3:**
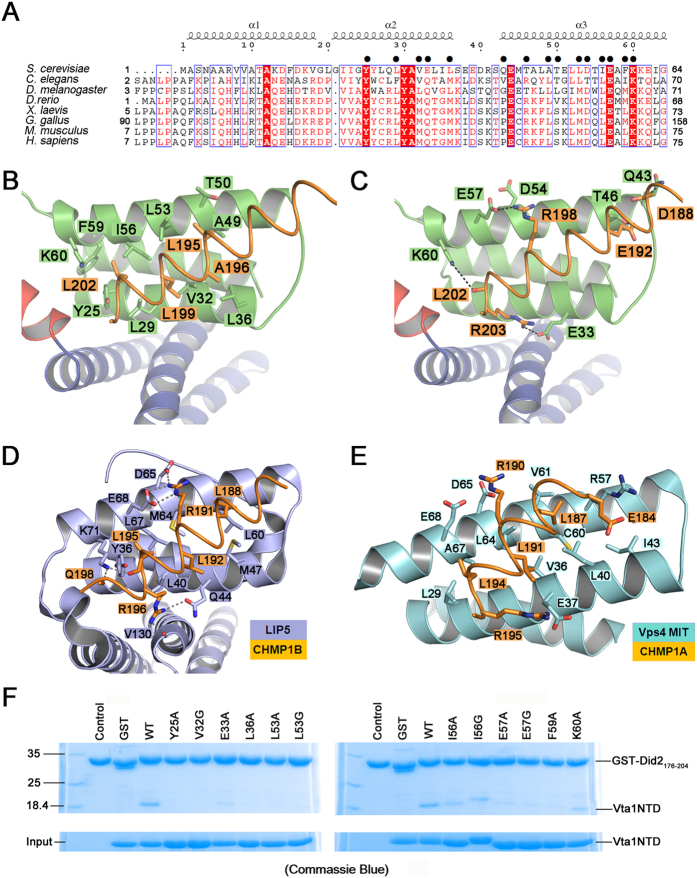
The conserved residues of Vta1NTD MIT1 domain interact with Did2_176–204_. (**A**) Structure-based sequence alignment of Vta1NTD MIT1 from different organisms. The secondary structures of Vta1NTD MIT1 were displayed on the top of the sequences. The residues in MIT1 involved in the interaction between Vta1NTD and Did2_176–204_ were marked by black dots. (**B**,**C**) The hydrophobic and electrostatic interactions observed in Vta1NTD-Did2_176–204_ complex, where Vta1NTD is displayed in green cartoon, while Did2 is in orange ribbon mode, respectively. (**D**,**E**) Hydrophobic and electrostatic interactions observed in LIP5NTD-CHMP1B complex (pdb code 4TXQ), and in Vps4-CHMP1A complex (pdb code 2JQ9), respectively. CHMP1B and CHMP1A were displayed in orange, LIP5NTD MIT1 and Vps4 MIT1 were displayed in grey-blue, respectively. All residues involved in the interaction were highlighted in stick. (**F**) GST pull-down assay of the Vta1NTD-Did2_176–204_ complex. GST or GST-tagged Did2_176–204_ was used to pull down wild type or mutant Vta1NTD as indicated. Proteins retained on the beads were analyzed by SDS-PAGE and visualized by coomassie-blue staining. The control GST-tagged Did2_176–204_ lane and the GST lane represented the cases where the gels were run in the absence of WT Vta1NTD. These two gels were run at different time.

Two major kinds of interactions between Vta1NTD MIT1 domain and Did2_176–204_ were observed (Fig. 3). One is predominantly hydrophobic, and is lined by the conserved and non-conserved residues Y25, L29, V32 and L36 in the 2^nd^ helix, and residues A49, L53, I56 and F59 in the 3^rd^ helix of Vta1NTD MIT1 (Fig. 3A and B). The side chains of the residues located at Did2_176–204_ helix α6′, including L195′, A196′, L199′ and L202′, are inserted into the groove of Vta1NTD helices 2/3. Did2_176–204_ L195′ side-chain has hydrophobic interaction with Vta1NTD A49, L53 and T46 side-chains, as residue L188 works in complex LIP5NTD-CHMP1B and LIP5NTD-CHMP1B-CHMP5 (Fig. 3D)^36^, and residue L187 functions in complex Vps4A-CHMP1A MIM1 (Fig. 3E)^20^. Did2_176–204_ A196′ has hydrophobic interactions with residue L36 of Vta1NTD (the distance is 2.3 Å between their methyl groups). Did2_176–204_ L199′ side-chain methyl group has hydrophobic interactions with Vta1NTD L29 and V32 methyl groups (the distances between them are 2.3 Å), corresponding to residue L191 functioning in complex Vps4A-CHMP1A MIM1^20^, and residue L192 working in complexes LIP5NTD-CHMP1B and LIP5NTD-CHMP1B-CHMP5^36^. In addition, Did2_176–204_ L202′ side-chain also has hydrophobic interaction with Vta1NTD Y25, L29, V32, L53, I56, F59 and the side-chain of K60, similar to the observation of the residue L194 in complex Vps4A-CHMP1A MIM1^20^, and residue L195 in complex LIP5NTD-CHMP1B and LIP5NTD-CHMP1B-CHMP5^36^. Besides these hydrophobic interactions, complementary salt bridges are further formed by two of the adjacent conserved Did2_176–204_ residues (R198′and R203′) (Fig. 3C). Did2_176–204_ R198′ forms a salt bridge with residues D54 and E57 in the 3^rd^ helix of Vta1NTD, while Did2_176–204_ R203′ has static electric interactions with negative side-chain of E33 in the 2^nd^ helix of Vta1NTD. These interactions were also observed in the complex structure of LIP5NTD-CHMP1B and LIP5NTD-CHMP1B-CHMP5, corresponding to electric interaction between R191′ in CHMP1B and E68 in LIP5NTD, and hydrogen interaction between R196′ in CHMP1B and Q44 in LIP5NTD^36^. These two major type of interactions were also confirmed by our ITC experiment, with negative ΔH (−2.7 Kcal mol^−1^) which can be attributed to the hydrogen-bond interaction as well as ionic interactions, and with positive ΔS (13.1 cal mol^−1^ deg^−1^) mainly derived from hydrophobic interactions in this case.

Mutations were introduced to these observed binding sites to test the importance of the residues to the overall stability of the complex. As shown in Fig. 3F, *in vitro* GST pull-down experiments demonstrate that all the single alanine or glycine substitutions of Vta1NTD residues Y25, E33, L36, L53, I56, E57, F59 and K60 have obvious effects on the Did2_176–204_ binding, confirming the energetic importance of all those residues. Consistent with the relatively small buried interface area of Vta1NTD-Did2 complex (compared to the surface area at the interface of Vta1NTD-Vps60_128–186_, approximately 3608 Å^2^), each single site mutation dramatically decreases all Did2_176–204_ binding, so that the binding affinities of all Vta1NTD variants to Did2_176–204_ were non-detectable.

### Did2 interacts with Vta1NTD in a classic MIM1 mode

**Figure 4:**
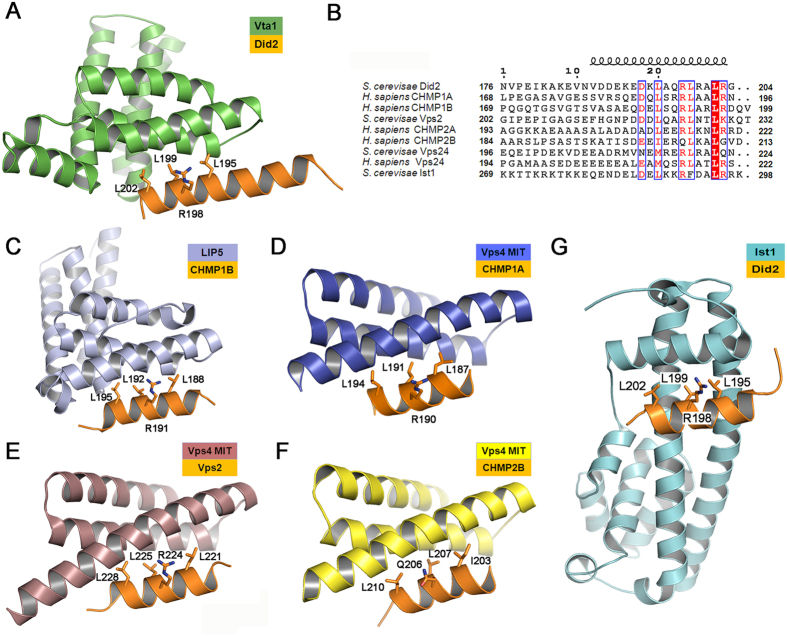
The conserved residues of Did2_176–204_ interact with Vta1NTD in MIM1 mode. (**A**) Ribbon representation of Vta1NTD-Did2_176–204_ complex. (**B**) Sequence alignments of Did2_176–204_ containing helix α6′ with conserved MIM1 motif from various organisms. The helix conformation was displayed on the top of the sequences based on free Did2_176–204_ structural calculation. (**C**–**F**) Ribbon representations of LIP5-CHMP1B complex (pdb code 4TXQ), Vps4 MIT-CHMP1A MIM1 complex (pdb code 2JQ9), Vps4 MIT-Vps2 MIM1 complex (pdb code 2V6X), and Vps4B MIT-CHMP2B complex (pdb code 2JQK); (**G**) Ribbon representation of Ist1NTD-Did2 complex (pdb code 3GGZ); In (**A**,**C**–**G**), all MIM1 domains were displayed in orange cartoon mode, and the conserved residues involved in the interactions were displayed in stick mode.

The MIT domain is a versatile protein-protein interaction domain identified in proteins that have a role in vesicle trafficking, including Vps4, Vta1, AMSH and UBPY, where they mediate interaction within the ESCRT-III complex^41^. The MIT domain recognizes sequence motifs called the MIMs primarily within the ESCRT-III subunits. It has been implicated that the interaction between MIT and MIM acts in regulating the disassembly of ESCRT-III as well as targeting specific proteins to the site of ESCRT functions. As we summarized in previous report^33^, five types of MIM (MIM1, MIM2, MIM3, MIM4 and MIM5) in total were reported to bind to different sites on the MIT domain^19^,^20^,^21^,^22^,^23^,^24^. Among them, MIM1 motif includes a sequence-conserved amphipathic helix (D/E)xxLxxRLxxL(K/R) along the groove between MIT helices α2 and α3 observed in the complexes Vps4-Vps2_183–232_^19^, Vps4-CHMP2B^19^,^20^, LIP5-CHMP1B^36^ and Vps4-CHMP1A_180–196_^20^ (Fig. 4A–F). MIM2 motif is a proline-rich sequence L_170_P(E/D)VP_174_ and R_183_xLxPxLPxPP_193_ along the groove between MIT helices α1 and α3 found in complex Vps4-CHMP6_168–179_^21^ and Saci1372-Saci1337_183–193_^22^, respectively. MIM3 motif is a highly specific mode along the groove between MIT helices α1 and α3 found in complex Spastin MIT-CHMP1B_148–196_^23^, but with a twice interface as large as that of MIT of the Vps4-CHMP complex. MIM4 motif is a mainly polar sequence E_203_xxxExxϕxxϕxxRLxTLR_221_ along a groove made up by helices 3 (Vps4 MIT helix 2) and 4/5 (Vps4 MIT helix 3) identified in complex AMSHΔC-CHMP3ΔN^24^. MIM5 was found in Vta1NTD-Vps60 or LIP5NTD-CHMP5 complex^33^,^35^. Vps60 or CHMP5 MIM5 sequence (residues 140–186) forms two helices (α4′ and α5′), and binds two surfaces made up by helices 5, 7 (Vps4 MIT helices 1, 3) and helices 6, 7 (Vps4 MIT helices 2, 3) of the Vta1/LIP5 MIT2 domain. The Vta1/LIP5 MIT2-Vps60/CHMP5 MIM5 contacts are a mixture of polar and hydrophobic interactions as the same as the case of Spastin MIT-MIM3.

On one hand, Vta1NTD MIT1 derived from *S. cerevisiae* has a little higher sequence similarity with the other organisms than the Did2_176–204_ sequence, as shown in Figs 3A and 4B. The conserved and non-conserved hydrophobic residues L29, V32, L36, L53 and I56 (which present an overall hydrophobic surface), and hydrophilic residues E33, T46, D54, E57 and K60 in Vta1NTD MIT1 play more important roles in the Vta1-Did2 interactions. These residues correspond to the residues Y34, L37, M41, L64, A67, D38, R57, E68 and K71 in Vps4 MIT domain, all of which are involved in its interactions with CHMP1A MIM1 (Fig. 4E). On the other hand, in Vta1NTD-Did2_176–204_ complex structure, the MIM region utilizes conserved hydrophobic residues L195′, L199′, and L202′ and hydrophilic residues R198′, R203′ to interact with MIT. These residues within Did2_176–204_ helix α6′ in the interface make up of an amino acid sequence as E_192_′xxL_195_′xxR_198_′L_199_′xxL_202_′R_203_′, nearly identical to CHMP1A_180–196_ MIM1 motif (D/E)xxLxxRLxxL(K/R)^19^,^20^. Thus, Did2 interacts with Vta1NTD through a classic MIM1 mode. Interestingly, although the binding mode of Did2 and Vta1NTD resembles that of LIP5NTD-CHMP1B, the extent of their further stimulation for Vps4 activity diverge from each other, which suggests different mechanism for Did2 or CHMP1B involved in MVB pathway.

In addition, as shown in Fig. 4G, the crystal structure of Ist1NTD-Did2 MIM1 complex indicated that Did2 MIM1 interacts with the groove made up by Ist1NTD helices α1, α3 and α5 through hydrophobic residues L195′, L199′, L202′ and positively charged residues R198′ and R203′ in helix α6′^42^. This observation suggested that Did2 MIM1 could not simultaneously interact with Vta1NTD and Ist1NTD. Moreover, the binding affinity (*K*_D_) of Ist1 to Did2 MIM1 is close to 1 μM, much stronger than that (*K*_D_ = ~39 μM) of Vps4 MIT domain binding to Did2 MIM1, and that (*K*_D_ = ~28 μM) Vps4 MIT domain binding to Vps2 MIM1 domain, and that (*K*_D_ = 12.8 μM) of Vta1NTD binding to Did2 MIM1. This observation reveals that Did2 MIM1 may prefer bind to Ist1NTD due to stronger binding affinity than that to Vta1NTD, and that the interaction between MIT and MIM1 domains in ESCRT-III system is not significantly specific. This analysis may interpret why MIT domain can interact with different subunits in ESCRT-III containing MIM1 domain.

### Either Did2 or Vps60 enhances Vta1 stimulation of Vps4 in a specifically indirect manner

The dynamic assembly and disassembly of the ESCRT-III polymer play a critical role in ESCRT-mediated membrane deformation events, and the alterations of Vps4 ATPase activity. To address how Vps60 and Did2 binding enhance Vta1 stimulation of Vps4 ATPase activity, two models were previously presented^27^. One is that their binding to MIT domains results in the conformation changes of Vta1; the other is that the interaction between Vta1 and Did2 or Vps60 increases the local concentration of Vta1–Vps4 *in vitro*. It was reported that removal of the two Vta1 tandem MIT domains (Vta1_165–330_) does not enhance the basal activation of Vps4 by Vta1, implying that Vta1 MIT domains does not autoinhibit Vps4 activation^27^. The NMR structures of complex Vta1NTD-Vps60_128–186_^33^,^34^,^35^ and Vta1NTD-Did2_176–204_ provided evidences that the Vps60 or Did2-binding did not induce overall conformational changes in the N-terminus of Vta1. These observations suggested that either Did2 or Vps60 did not allosterically regulate Vta1NTD and thus could not potentiate its ability to directly activate Vps4. Recently, at C-terminus of Vta1, a novel short amino acid sequence, called as Vps4 stimulatory element (VSE), was identified to be released to stimulate Vps4 ATPase activities, upon Vta1NTD interacting with ESCRT-III Did2 or Vps60^43^. VSE activity is auto-inhibited in a manner dependent upon the unstructured linker region, which joints the N-terminal MIT domains and the C-terminal VSL domain. Thus, although structural studies on Vta1NTD-Vps60_128–186_ and Vta1NTD-Did2_176–204_ provided no direct evidences of how Vps60 and Did2 function, Vps60 or Did2 binding to Vta1NTD might lead to further structural arrangement in the C-terminal domain of Vta1. Either Did2 or Vps60 enhances Vta1 stimulation of Vps4 in a specifically indirect manner.

In summary, we determined NMR solution structure of Vta1NTD-Did2_176–204_, which provided the molecular basis of how Did2 interacts with Vta1NTD. Structural comparison and sequence alignment suggest that Did2 binds to Vta1NTD in a classic MIM1 mode. Both Vps60_128–186_ and Did2_176–204_ stimulate Vps4 activities by releasing VSE through interaction with Vta1NTD.

## Methods

### Cloning, expression, and purification

DNA fragments encoding yeast Vta1 and Did2 were amplified from the *S. cerevisiae* genomic DNA. Vta1NTD and Did2_176–204_ were expressed in *Escherichia coli* BL21(DE3) using a modified pET28b vector with a SUMO protein inserted between a His_6_-tag and the Vta1NTD or Did2_176–204_ coding region, respectively. To correctly estimate the concentration of Did2_176–204_ during its purification, an extra residue tryptophan was inserted in the N-terminus of the peptide during constructing the plasmid. To obtain pure Vta1NTD, the His_6_-tagged SUMO-Vta1NTD was purified by Ni^2+^-NTA affinity chromatography (GE Healthcare, USA) following standard procedures. ULP1 protease was then added to remove the His_6_-SUMO tag and the protein mixture was passed over a second Ni^2+^-NTA column and was further purified by anion exchange chromatography on a Resource Q column (GE Healthcare, USA). The Vta1NTD variants were purified in the same way as native proteins. To prepare pure Did2_176–204_, the His_6_-tagged SUMO Did2_176–204_ was first purified by Ni^2+^-NTA affinity chromatography and by anion exchange chromatography on a Resource Q column, respectively. Then ULP1 protease was added to remove the His_6_-SUMO tag, and the protein mixture was passed over a gel-filtration chromatography Superdex 75 column (GE Healthcare, USA). The concentration of Did2_176–204_ was finally obtained from the absorbance at 280 nm with an absorption coefficient of 5500 M^−1^cm^−1^. The peptide solution was lyophilized for future usage.

For isotope labeling NMR sample (either Vta1NTD or Did2_176–204_), M9 minimal medium was used supplemented with ^15^NH_4_Cl (Cambridge Isotope Laboratories, USA) or ^15^NH_4_Cl and ^13^C-glucose (Cambridge Isotope Laboratories, USA), or ^15^NH_4_Cl, ^13^C-glucose and 70% D_2_O (Cambridge Isotope Laboratories, USA).

### NMR sample preparation and data collection

Differentially labeled complex samples in NMR buffer (25 mM sodium phosphate pH 7.0, 100 mM NaCl, 5 mM dithiothreitol-d_10_ (DTT), 0.02% NaN_3_, 10% D_2_O), were prepared as follows: 1) 1.5 mM uniformly ^15^N-/^13^C double labeled or ^15^N-/^13^C-/70% ^2^H triple labeled Vta1NTD plus 1.8 mM unlabeled Did2_176–204_; 2) 1.5 mM uniformly labeled ^15^N-/^13^C-labeled Did2_176–204_ in complex with 1.8 mM unlabeled Vta1NTD. All NMR experiments were performed at 20 °C on a Varian Unity Inova 600 NMR spectrometer (with cryo-probe) equipped with triple resonances and pulsed field gradients, or on a Bruker Avance III-800 MHz NMR spectrometer (with cryo-probe) equipped with four channels and z-axis pulsed-field gradient. The standard suite of experiments for assigning the ^1^H, ^13^C and ^15^N backbone and side chain chemical shifts of ^13^C and ^15^N double labeled Vta1NTD in complex with unlabeled Did2_176–204_, or of ^13^C and ^15^N double labeled Did2_176–204_ in complex with unlabeled Vta1NTD, and for the collection of NOE-based distance restraints were measured^44^,^45^, including the two-dimensional (2D) ^13^C-edited HSQC in both aliphatic and aromatic regions, and ^15^N-edited HSQC; the three-dimensional (3D) HNCA, HNCO, HN(CO)CA, HNCACB, CBCA(CO)NH, ^15^N-resolved HSQC-TOCSY, HCCH-TOCSY in both aliphatic and aromatic regions, ^15^N-resolved HSQC-NOESY, ^13^C-resolved HSQC-NOESY for both aliphatic and aromatic resonances, 2D (H_β_)C_β_(C_γ_C_δ_)H_δ_ and (H_β_)C_β_(C_γ_C_δ_C_ε_)H_ε_ spectra for correlation of C_β_ and H_δ_ or H_ε_ in aromatic ring used in aromatic protons assignment^46^. The intermolecular NOEs between isotope labeled Vta1NTD or Did2_176–204_ peptide and unlabeled Did2_176–204_ peptide or Vta1NTD were obtained by analyzing 3D ^13^C-F1 edited, ^13^C/^15^N-F3 filtered NOESY spectra, respectively.

For assignment of NMR signals belonging to free Did2_176–204_, the isotope ^15^N-labeled Did2_176–204_ and unlabeled Did2_176–204_ were used at the concentration of 1.0 mM in NMR buffer. 2D ^1^H-^1^H TOCSY and NOESY, as well as 3D ^15^N-edited HSQC-TOCSY experiment, were acquired at 20 °C only on the Varian Unity Inova 600 NMR spectrometer (with cryo-probe, as mentioned above).

All spectra were processed with the program NMRPipe^39^ and analyzed with the Sparky 3 software^47^. The ^1^H chemical shifts were referenced to 2,2-dimethylsilapentane-5-sulfonic acid (DSS), and the ^13^C- and ^15^N-resonances were indirectly referenced DSS.

### NMR structure determination

The structural calculations of free Did2_176–204_ and of the complex Vta1NTD-Did2_176–204_ were carried out using a standard simulated annealing protocol implemented in the program XPLOR-2.19 (NIH version). The inter-proton distance restraints derived from NOE intensities were grouped into three distance ranges 1.8–2.9 Å, 1.8–3.5 Å and 1.8–6.0 Å, corresponding to strong, medium and weak NOEs, respectively. The dihedral angles phi and psi were derived from the backbone chemical shifts (HN, HA, CO, CA) by the program TALOS^39^,^40^. Slowly exchanging amide protons, identified in the 2D ^15^N-HSQC spectra recorded after a H_2_O buffer was exchanged to a D_2_O buffer, were used in the structure calculated with the NOE distances restraints to generate hydrogen bonds for the final structure calculation, as done in the literature^48^. A total of ten iterations^49^ structures in the initial eight iterations were performed. 100 structures were computed in the last two iterations, 20 conformers with the lowest energy are used to represent the 3D structures. In the ensemble of the simulated annealing 20 structures, there was no distance constraint violation more than 0.3 Å and no torsion angle violation more than 3°. The final 20 structures of the complex Vta1NTD-Did2_176–204_ or free Did2_176–204_ with lowest energy were evaluated with the program PROCHECK-NMR and PROCHECK^50^ and summarized in Table 1. All figures were generated using the program PyMOL (http://pymol.org/) and MOLMOL^49^.

### Isothermal titration calorimetry (ITC) assay

To obtain a direct binding affinity between Vta1NTD and Did2_176–204_ peptide, solution of about 0.1 mM Vta1NTD was titrated with 2.0 mM Did2_176–204_ peptide using iTC-200 microcalorimeter (GE healthcare, USA) at 25 °C. The protein and peptide were exchanged to a buffer containing 25 mM sodium phosphate, pH 7.0 and 50 mM NaCl by gel-filtration chromatography, centrifuged to remove any particulates, and degassed. The accurate concentrations of Vta1NTD and Did2_176–204_ concentration were determined using their A^280^ coefficient constants. The obtained data were fitted by a nonlinear least squares approach to the ‘one set of sites’ binding model from Microcal Origin software, which yielded the association constant (*K*_a_), stoichiometry of binding (n), and the thermodynamic parameters, enthalpy change of binding (ΔH), entropy change of binding (ΔS) and free energy change of binding (ΔG). The ITC experiment was repeated at least two times for validity.

### GST pull-down experiments

The experiments were performed following standard procedures in buffer containing 25 mM Tris-HCl (pH 8.0), 150 mM NaCl, and 5 mM 2-mercaptoethanol. Purified WT Vta1NTD and its mutants were incubated with either GST alone or GST-tagged Did2_176–204_ immobilized on glutathione agarose beads for 3 h at 4 °C. The beads were then washed extensively with above buffer three times, and bound proteins were separated on SDS-PAGE and visualized by Coomassie-blue staining. The pull-down experiments were repeated three times with the similar results. The representative results were shown in Fig. 3F. The control GST-tagged Did2_176–204_ alone lane and the GST alone lane indicated the cases where the gels were run in the absence of WT Vta1NTD. The two gels were run at different time.

## Additional Information

**How to cite this article**: Shen, J. *et al*. NMR studies on the interactions between yeast Vta1 and Did2 during the multivesicular bodies sorting pathway. *Sci. Rep.* **6**, 38710; doi: 10.1038/srep38710 (2016).

**Publisher's note:** Springer Nature remains neutral with regard to jurisdictional claims in published maps and institutional affiliations.

## Figures and Tables

**Table 1:** Experimental restraints and structural statistics for Vta1NTD-Did2_176–204_ complex and free Did2_176–204_.

Experimental restraints and structural statistics		
NMR distance and Dihedral Constraints	Vta1NTD-Did2_176–204_	free Did2_176–204_
Distance restraints from NOEs		
Intra-molecular		
Total	1811	191
Intra-residue (i-j = 0)	778	102
Sequential (|i-j| = 1)	487	78
Medium range (1<|i-j|≤5)	434	11
Long range (|i-j|>5)	112	0
Inter-molecular	36	
Hydrogen bonds	236	26
Dihedral restraints	390	
φ	195	
ψ	195	
**Structural statistics**^a^		
R.m.s Deviations versus the mean structure (Å)		
All backbone atoms	2.29 ± 0.53	3.14 ± 0.90
All heavy atoms	2.52 ± 0.49	3.71 ± 0.83
Backbone atoms (secondary structure)	0.82 ± 0.17	0.15 ± 0.06
Heavy atoms (secondary structure)	1.14 ± 0.15	0.88 ± 0.09
R.m.s. deviations from the experimental restraints		
NOE distances (Å)	0.034 ± 0.0007	0.24 ± 0.008
Dihedral angles (°)	0.53 ± 0.032	
R.m.s. deviations from idealized geometry		
Bonds (Å)	0.002 ± 0.00003	1.56 ± 0.00009
Angles (°)	0.28 ± 0.0068	0.25 ± 0.012
Impropers (°)	0.27 ± 0.006	0.21 ± 0.009
**Ramachandran analysis**		
Residues in most favored regions	97.8%	93.1%
Residues in additionally allowed regions	2.1%	4.0%
Residues in generously allowed regions	0.1%	2.0%
Residues in disallowed regions	0.0%	0.0%

^a^Structural statistics was calculated from 20 lowest-energy XPLOR-NIH structure.
